# Molecular Regulation of Phenylpropanoid and Flavonoid Biosynthesis Pathways Based on Transcriptomic and Metabolomic Analyses in Oat Seedlings Under Sodium Selenite Treatment

**DOI:** 10.3390/biology14091131

**Published:** 2025-08-26

**Authors:** Jianxia Ma, Xiaozhuo Wu, Huichun Xie, Guigong Geng, Feng Qiao

**Affiliations:** 1 Key Laboratory of Tibetan Plateau Medicinal Plant and Animal Resources, School of Life Sciences, Qinghai Normal University, Xining 810008, China; majianxia0926@163.com (J.M.); xiaozhuo0623@163.com (X.W.); 2025090@qhnu.edu.cn (H.X.); 2 Academy of Plateau Science and Sustainability, Qinghai Normal University, Xining 810008, China; 3 Qinghai South of Qilian Mountain Forest Ecosystem Observation and Research Station, Huzhu 810500, China; genggg-298@163.com; 4 National Forestry Grassland Qinghai Tibet Plateau Characteristic Forest and Grassland Germplasm Resources Protection and Utilization Engineering Technology Research Center, Xining 810008, China; 5 Academy of Agricultural and Forestry Sciences, Qinghai University, Xining 810016, China

**Keywords:** oat seedlings, Na_2_SeO_3_ treatment, transcriptome, metabolome, phenylpropanoid and flavonoid biosynthesis pathways

## Abstract

Selenium can be absorbed and utilized by plants, influencing their growth by altering their physiological metabolism. In this study, based on plant physiology methods, compared to the CK treatment, the height and leaf length of oat seedlings under the T0.02 (0.02 g/kg Na_2_SeO_3_) treatment significantly increased by 18.36% and 15.81%, respectively (*p* < 0.05). Under the T0.1 (0.1 g/kg Na_2_SeO_3_) treatment, the levels of malondialdehyde (MDA), proline, soluble sugar content, and peroxidase (POD) activity significantly increased (*p* < 0.05). However, the seedling height and leaf length under the T0.1 treatment significantly decreased by 33.24% and 23.25%, respectively. Additionally, the contents of chlorophyll a, chlorophyll b, and carotenoids, as well as ascorbate peroxidase (APX) activity and the superoxide anion radical generation rate (O_2_^−^) significantly decreased (*p* < 0.05). The total selenium, organic selenium, and inorganic selenium contents, as measured by the atomic fluorescence spectroscopy method, were also increased in oat seedling roots and leaves under T0.1 treatment (*p* < 0.05). Selenium had a high coefficient of mobility from root to leaf of 6.01 under T0.02 and 4.65 under T0.1 treatment, and from soil to leaf of 4.98 under T0.02 and 4.55 under T0.1 treatment. Through untargeted metabolomics, six differential phenylpropanoid compounds and 18 differential flavonoid compounds were found in oat seedlings. Based on transcriptomic analysis of oat seedlings, 29 DEGs associated with phenylpropanoid metabolism and 13 DEGs related to flavonoid biosynthesis were identified. Over 60% of the genes (25/42) in the phenylpropanoid and flavonoid biosynthesis pathway were associated with the accumulation of about 74% (20/27) of the compounds in oat leaves. Based on transcriptomic and metabolomics analysis, there were nine major genes (including *PAL1*, *PAL4*, *CHS2*, *PAL7*, *POD3*, *PAL6*, *CCR1*, *CCR4*, *POD4*) modulating the metabolism of phenylpropanoid and flavonoid biosynthesis pathway. This study offers novel insights and genetic resources for exploring the mechanisms underlying plant responses to selenium treatment, thereby further enhancing selenium tolerance in plants.

## Simple Summary

This study investigates the effects of varying concentrations of sodium selenite (Na_2_SeO_3_) on the growth of oat seedlings, as well as the biosynthesis pathways of phenylpropanoids and flavonoids, utilizing transcriptomics, metabolomics, and physiological and biochemical analyses. The results indicate that the T0.02 (0.02 g/kg Na_2_SeO_3_) treatment significantly promoted the growth of oat seedlings. As the concentration of Na_2_SeO_3_ increased, levels of proline and soluble sugars increased significantly, while pigment content, peroxidase activity, and the generation rate of superoxide anions decreased significantly. The selenium content in the roots and leaves of oat seedlings increased significantly under the T0.1 treatment (*p* < 0.05). Additionally, six differential metabolites and 29 differential genes related to the phenylpropanoid synthesis pathway were identified, along with 18 differential metabolites and 13 differential genes associated with the flavonoid synthesis pathway. By integrating transcriptomic and metabolomic analyses, nine key genes (including *PAL1*, *PAL4*, *CHS2*, *PAL7*, *POD3*, *PAL6*, *CCR1*, *CCR4*, *POD4*) regulating the metabolism of the phenylpropanoid and flavonoid biosynthesis pathways were screened. This study offers new insights and genetic resources for exploring the response mechanisms of oats to selenium treatment.

## 1. Introduction

Selenium is an essential trace nutrient for organisms [1], possessing various biological functions [2,3,4], including the prevention of cardiovascular and cerebrovascular diseases, anti-aging effects, antioxidant properties [5], anti-cancer effects [6], and the enhancement of immunity [7]. Additionally, selenium promotes the growth and development of plants, acting as an antioxidant or stimulant in a dose-dependent manner to protect them from various abiotic stresses [8]. Plants serve as the primary source of selenium in the food chain, and the concentration and availability of selenium in plants are influenced by soil conditions and environmental factors [9]. Appropriate selenium concentrations in soil can enhance selenium content in plants, promote growth, improve stress resistance, increase yield, and enhance fruit quality [10]. Research has demonstrated that an appropriate amount of selenium can enhance the growth and development of *Salvia miltiorrhiza*, improve its antioxidant capacity [11], and play a significant role in the photosynthetic efficiency and mineral element absorption in crops such as rice [12] and wheat [13]. Furthermore, low concentrations of exogenous selenium can increase the nutritional and medicinal value of these crops by regulating the accumulation of secondary metabolites. For instance, it can elevate the content of terpene lactones in *Ginkgo biloba* leaves [14] and enhance the flavonoid content and biosynthesis of phenolic acids in *Brassica Oleracea Var. Capitata* L. [15]. However, excessive selenium concentrations can inhibit plant growth and development, potentially leading to plant death, which adversely affects yield and quality. High selenium treatment impairs the normal physiological functions of *Brassica napus* by reducing ascorbic acid levels and the activity and expression of catalase, glutathione reductase, and dehydroascorbate reductase, resulting in a decline in biomass and yield [16]. Additionally, high selenium levels decrease the survival rate of *Atractylodes macrocephala*, impacting its yield [17]. These studies provide a foundation for understanding plant responses to both suitable and high concentrations of selenium.

The phenylpropanoid biosynthetic pathway generates over 8000 metabolites and serves as the primary source of defensive secondary metabolites, including flavonoids, lignin, and salicylic acid [18]. This pathway is essential for plant growth and development, as well as their interactions with the environment [19]. Its metabolites can scavenge reactive oxygen species (ROS), thereby preventing damage to cellular morphology, structure, and physiological metabolic functions [20]. Additionally, these metabolites enhance plant disease resistance and stress tolerance, enabling plants to defend against external physical and biological invasions [18]. The flavonoid biosynthesis pathway, which is derived from the phenylpropanoid pathway, features a C6-C3-C6 benzene ring structure [21,22] and can be classified into various types based on their chemical structures, including flavones, flavonols, flavanones, flavanols, isoflavones, chalcones, and anthocyanins [23,24]. Flavonoids play significant physiological roles in plant tolerance responses to abiotic stresses, such as ultraviolet radiation, extreme temperatures, salinity, drought, and heavy metals [25]. Furthermore, flavonoids are prevalent in the human diet, exhibiting antioxidant, antimicrobial, and anti-inflammatory properties [26].

Oat (*Avena sativa* L.), an annual cereal crop belonging to the Poaceae family [27], is recognized for its advantages, including tolerance to poor soils, strong adaptability, high nutritional value, and tall plant stature. As a dual-purpose crop for both human consumption and animal feed [28], oat is extensively cultivated in cold, high-altitude regions. It can adapt to various environmental stresses, including drought, salinity, and pathogen attacks, undergoing morphological and physiological changes to acclimate to stress conditions [29,30]. Compared to rice, wheat, and other forage crops, oat exhibits a higher capacity for stress resistance [31,32,33]. It is rich in lipids and contains a significant amount of polyphenolic compounds, including bioactive substances such as caffeic acid, coumaric acid, protocatechuic acid, flavonoids, isoflavones, and lignans [34,35]. However, tolerance to abiotic stress is a quantitative trait controlled by multiple genes [36,37]. In order to better understand the physiological responses and molecular regulation mechanisms of selenium in oat seedlings, it is necessary to study them through physiological metabolism, metabolomics, and transcriptomics.

This study investigates the effects of various concentrations of selenium in oat seedlings by measuring their morphological, physiological, and biochemical indicators. Through transcriptomic and metabolomic analyses, we explored the response mechanisms of oat seedlings to exogenous selenium treatment, with particular emphasis on the roles of the phenylpropanoid and flavonoid biosynthesis pathways. Furthermore, key genes and metabolites involved in this pathway under selenium treatment were screened. This research provides valuable insights into the impact of selenium stress on plants and the mechanisms of selenium tolerance, while also offering new perspectives for the high-quality and high-yield cultivation of oats.

## 2. Materials and Methods

### 2.1. Experimental Materials and Treatments

The oat seeds employed in this study were provided by the Crop Research Institute of the Qinghai Academy of Agriculture and Forestry Sciences. Sodium selenite (Na_2_SeO_3_, analytical grade, Qinghai Lainer Biotechnology, Xining, China) was utilized as the source of selenium. For the pot experiment, plastic pots measuring 25 cm × 12 cm × 12 cm were used. Culture Substrate Treatment: The sandy soil was sieved (The sterilized sand is essentially a collection of natural mineral particles that have been sterilized, mainly composed of silica, feldspar, mica and other silicate minerals and a small amount of metal oxides, containing air and trace moisture, and free of live microorganisms and organic impurities), autoclaved at 120 °C for 20 min, air-dried, and subsequently mixed with vermiculite at a 1:1 volume ratio before being placed into pots, each containing 720 g of the substrate weight per pot. Oat Seed Treatment: Uniformly sized seeds were selected, rinsed 3–5 times with tap water to eliminate surface dust and impurities, soaked in 75% ethanol for 5 min, and then rinsed three times with ultrapure water. The sterilized oat seeds were evenly sown in plastic pots filled with a mixed substrate, with 25 seeds per pot at a depth of approximately 2 cm. The pots were cultivated under conditions where the temperature was maintained at 25 ± 3 °C, humidity was kept between 40% and 50%, the photoperiod was set to 12 h per day, and the light intensity ranged from 10,000 to 15,000 Lux. The pots received irrigation with 500 mL of 1 × Hoagland [38] nutrient solution every three days, and the growth of the seedlings was observed and recorded daily. After one week of growth, the oat plants exhibited robust health with no signs of pests or diseases, averaging a height of approximately 5 cm. From each pot, 20 uniformly growing seedlings were selected and treated with Na_2_SeO_3_ solutions at varying concentrations: 0.01, 0.02, 0.05, 0.075, and 0.1 g·kg^−1^, in addition to a control treatment. The solutions were prepared using 1 × Hoagland nutrient solution, and each concentration was replicated three times, resulting in a total of 18 pots. The control group received an equivalent volume of 1 × Hoagland nutrient solution. The Na_2_SeO_3_ nutrient solution was applied every three days. After a 15-day cultivation period, oat seedlings from both the control group (CK) and those subjected to low selenium concentration T0.02 (0.02 g·kg^−1^) and high selenium concentration T0.1 (0.1 g·kg^−1^) were selected, packaged, and randomly divided into three distinct groups. One group was utilized as fresh samples on the day of sampling to determine physiological indicators, including plant height and root length. The second group was inactivated at 105 °C and dried to a constant weight at 80 °C, with the dry weight of the leaf measured on the day of sampling. The final set of samples, which included both soil and plant specimens, was rapidly frozen in liquid nitrogen and subsequently stored in a −80 °C ultra-low temperature freezer. This process was carried out to facilitate the determination of relevant biochemical indicators, the measurement of selenium content (all measurements were completed within one week of sampling), and the sequencing of transcriptomic and metabolomic data.

The phenotypes, including seedling height, root length, leaf length, leaf width, and leaf dry weight, were analyzed based on the average values and standard deviations of 15 individual plants under the CK, T0.02, or T0.1 treatment. The physiological indicators, including contents of chlorophyll a, chlorophyll b, chlorophyll a + b, carotenoid, MDA, proline, soluble sugar (SS), soluble protein (SP), H_2_O_2_, O_2_^−^, and the activities of APX, SOD, and POD, were measured with 3 biological replicates.

### 2.2. Determination of Physiological and Biochemical Indices

Daily observations of oat seedling growth were conducted until the 15th day, at which point 15 uniformly grown oat seedlings were randomly selected. 

Plant Height Measurement: A ruler with a precision of 0.5 mm was used to measure the height of the plant from its base, near the roots, to the highest growth point while the plant was in its natural growth state. The measurer’s line of sight should be aligned with the scale markings to prevent reading bias that may arise from viewing the ruler from above or below. The root length was determined through a complete excavation method. To acquire the root system, the bottom of the pot was gently tapped to remove the seedlings along with the substrate. The roots were then rinsed slowly with clean water, utilizing a 0.5 mm mesh sieve to prevent the loss of fine roots. After rinsing, the roots were spread flat on white filter paper and gently combed with a soft brush to avoid tangling. The longest 1–3 adventitious roots were selected, and the straight-line distance from the root base (the point of connection to the stem) to the root tip was measured and averaged. The total root length was obtained by measuring the length of all adventitious roots individually and summing them. The line of sight was perpendicular to the ruler scale reading. Fully expanded leaves from the middle part of the plant were selected, measuring 1–2 leaves per plant for leaf length and width. Leaf length was measured by placing a ruler (accuracy 0.5 mm) close to the back of the leaf and reading the length along the direction of the main vein; if the leaf was slightly curved, the arc length was measured in its natural state. The line of sight was level with the scale line corresponding to the tip of the leaf. Leaf width was measured using a vernier caliper (accuracy 0.1 mm) at the widest part, ensuring not to press the leaf to prevent width compression. The line of sight was level with the scale lines corresponding to the edges on both sides, ensuring that the reading was the actual vertical distance. Determination of dry weight of leaves: The seedling leaves were first placed in an oven at 105 °C for 30 min to inactivate enzymes. Subsequently, the seedlings were transferred to an oven set at 80 °C to achieve a constant dry weight, measured and recorded with an electronic balance (1/10,000, ESJ200-4B, Shenyang Longteng Electronic Co. Ltd., Shenyang, China). The data for each potted plant were represented by the averages of multiple measurements of plant height, root length, leaf length, leaf width, and dry weight of the leaf.

Chlorophyll content determination involved the following steps: First, 0.2 g of fresh leaves was taken and added to a measured quantity of quartz sand, calcium carbonate powder, and 95% ethanol. The mixture was ground into a homogeneous slurry and transferred into a small test tube. Then, 95% ethanol was added to achieve the desired volume, and the mixture was allowed to rest before centrifuging it. The blank control was 95% ethanol, and absorbance values were measured at wavelengths of 665 nm, 649 nm, and 470 nm [39]. MDA content determination involved weighing and slicing the leaves, which were subsequently ground into an extract solution. This solution was then shaken with thiobarbituric acid. Following thorough mixing, the solution was boiled, cooled, and centrifuged. The absorbance values were measured at wavelengths of 450 nm, 532 nm, and 600 nm using the supernatant. [40]. The soluble sugar content was extracted using a boiling water bath with 80% ethanol. The Anthracene colorimetric method was employed at a wavelength of 620 nm, utilizing an ultraviolet-visible spectrophotometer for measurement [41]. Determination of proline content involved taking cut leaves and treating them with a sulfosalicylic acid solution. The leaves were ground, followed by an extraction after boiling in a water bath. A color developer was added, and the mixture was heated in a water bath. Following the standard curve production method, a toluene extraction was performed, and the absorbance was measured at a wavelength of 520 nm. The proline content was then calculated using the standard curve [42]. Enzyme activity assay: The seedling leaves were placed in a pre-cooled mortar. The extraction medium was added, and the mixture was ground into a homogeneous slurry while an ice bath was maintained. After adjusting the volume, the slurry was centrifuged to separate the supernatant, which constituted the crude enzyme solution. Subsequently, the enzyme activities were assessed using the nitrogen blue tetrazole photoreduction method to determine superoxide dismutase (SOD) activity [43], the guaiacol chromogenic method for peroxidase (POD) activity [44], and the hydrogen oxidation method for ascorbate peroxidase (APX) activity [45]. Soluble protein content determination: Fresh leaves were weighed prior to grinding. The sample extraction solution for testing was obtained after centrifugation. The absorption at a wavelength of 595 nm was determined after mixing the sample with Mas Bright Blue G-250. The photometric value was calculated using a standard curve [46]. O_2_^−^ generation rate determination: Centrifugation of Plant Tissue Grinding. The solution to be tested was prepared by adding phosphoric acid buffer and hydroxylamine hydrochloride to the sample at a temperature of 25 °C. The mixture was maintained at this temperature for 1 h. Subsequently, p-aminobenzenesulfonic acid and α-naphthylamine were added, and the mixture was kept warm while being thoroughly mixed. Finally, the absorbance value was measured at a wavelength of 530 nm [47]. H_2_O_2_ content measurement: Acetone was added to the plant tissues. The extraction solution was milled and centrifuged, and the sample’s absorbance was measured at 560 nm using the xylene orange method, which was then used to calculate the content of hydrogen peroxide [48]. All reagents mentioned above were sourced from Qinghai Lainer Biotechnology in Xining, China.

### 2.3. Determination of Selenium Content

Sample Pretreatment: Soil samples were collected using the multi-point mixing method and subsequently reduced to approximately 50 g through quartering after thorough mixing. Plant residues and other impurities were meticulously removed, and the soil was air-dried naturally, avoiding direct sunlight to prevent selenium volatilization, with regular turning during the drying process. Following air-drying, the soil was ground using an agate mortar, sieved through a 100-mesh sieve (aperture 0.15 mm), and stored in polyethylene sealed bags for subsequent use. After harvesting the oat seedlings, the roots and leaves were separated. The roots were gently rinsed with deionized water 3–4 times, while the leaves were rinsed to eliminate surface dust. Excess moisture was absorbed using absorbent paper, and both roots and leaves were cut into pieces and dried in an oven at 60 °C until a constant weight was achieved. After drying, they were ground into powder using an agate mortar, sieved through a 60-mesh sieve (0.25 mm), and stored in polyethylene sealed bags for further use.

Total Selenium Determination: The total selenium determination method follows the original standard, specifically executing the first method outlined in GB/T 5009.93 [49]. A sample of 0.5 to 1 g of soil is weighed, and the soil matrix is completely destroyed through digestion with mixed acids, such as the HNO_3_-HClO_4_-HF system. After the acids have been expelled, the solution is diluted to volume with 5% HCl. For roots and leaves, a sample weighing 0.2 to 0.5 g is subjected to microwave digestion using HNO_3_ and H_2_O_2_. Following acid expulsion, the solution is also diluted to volume with 5% HCl. The selenium concentration in the solution is determined using atomic fluorescence spectrometry (Titan Instruments, Orchard Park, NY, USA, AFS-8220), and this measurement is converted to represent the total selenium content in the sample.

The determination of inorganic selenium is conducted in accordance with GB/T 5009.93, which outlines the method for determining selenium in foods using Hydride Generation Atomic Fluorescence Spectrometry (Method 1). For soil samples, the procedure involves weighing the sample and extracting it by shaking with 0.1 mol/L HCl or deionized water for a duration of 2 to 4 h. Following extraction, the sample is centrifuged and filtered, and the supernatant is collected. For root and leaf samples, the grinding of the sample is followed by extraction using 1 mol/L NH_4_Cl solution for 3 h, after which the sample is also centrifuged and filtered to obtain the supernatant. The extract is then directly measured using an atomic fluorescence spectrometer (Titan Instruments, AFS-8220), with the resulting value representing the inorganic selenium content. The organic selenium content is calculated as the total selenium content minus the inorganic selenium content.

### 2.4. Metabolite Extraction and LC-MS/MS Analysis

Oat seedling samples treated with CK, T0.02, and T0.1 were subjected to four biological replicates for each treatment concentration. Initially, the samples were vacuum freeze-dried, and 50 mg of each sample was weighed. Subsequently, 200 μL of pre-cooled 80% aqueous methanol solution was added along with two steel beads, and the mixture was homogenized and disrupted in a tissue disruptor at low temperature. Following this, an additional 800 μL of pre-cooled 80% aqueous methanol solution was added, the mixture was vortexed, sonicated in an ice bath for 20 min, and allowed to stand at −20 °C for 2 h. The samples were then centrifuged at 16,000 g at 4 °C for 20 min, and the supernatant was collected and dried using a high-speed vacuum centrifuge. The samples were redissolved in 100 μL of 50% aqueous methanol solution, centrifuged at 20,000 g at 4 °C for 15 min, and the supernatant was taken for mass spectrometry injection analysis. Throughout the analytical process, the samples were maintained at 4 °C in an autosampler. The separation of the samples was performed using the Shimadzu Nexera X2 LC30AD ultra-high-performance liquid chromatography (UHPLC) system equipped with an HSS T3 column (Shimadzu Corporation, Kyoto, Japan). The injection volume was set at 5 μL, the column temperature was maintained at 40 °C, and the flow rate was 200 µL/min. The mobile phases used for chromatography were A: 0.1% formic acid in water and B: acetonitrile. Each sample was analyzed in both positive ion (+) and negative ion (−) modes utilizing electrospray ionization (ESI). After UPLC separation, the samples were analyzed by mass spectrometry using the QTRAP 5500 mass spectrometer (Sciex, Framingham, MA, USA), with ionization conducted via the HESI source. The parameters for the QTRAP 5500 ESI source were as follows: Positive ion mode: Source Temperature 550 °C, Ion Source Gas1 (GAS1): 40, Ion Source Gas2 (GAS2): 50, Curtain Gas (CUR): 35, Ion Spray Voltage Floating (ISVF) 5500 V. Negative ion mode: Source Temperature 550 °C, Ion Source Gas1 (GAS1): 40, Ion Source Gas2 (GAS2): 50, Curtain Gas (CUR): 35, Ion Spray Voltage Floating (ISVF) −4500 V. The target ion pairs were detected using the MRM mode.

Principal Component Analysis (PCA) and Orthogonal Partial Least Squares Discriminant Analysis (OPLS-DA) were utilized for data reduction and regression, respectively. Qualitative results were derived from the HMDB and KEGG databases. The Variable Importance in Projection (VIP) value is usually calculated when using Partial Least Squares Discriminant Analysis (PLS-DA) or other multivariate statistical methods, which is used to measure the intensity and explanatory ability of the expression patterns of each metabolite on the classification discrimination of each group of samples, and to mine the biologically significant differential metabolites. The R language package ‘ropls’ was employed to perform OPLS-DA modeling, and 200 permutation tests were conducted to verify the reliability of the model. The Variable Importance in Projection (VIP) value of the model was calculated using multiple cross-validation. A combined approach incorporating the difference multiple, the *p*-value, and the VIP value of the OPLS-DA model was utilized to screen for differential metabolites. In this study, a screening criterion of VIP > 1 was implemented to preliminarily identify differential metabolites among the groups. Subsequently, univariate statistical analysis was performed to ascertain whether the metabolites exhibited significant differences. Metabolites that met the criteria of both multivariate statistical analysis (VIP > 1) and univariate statistical analysis (*p* value < 0.05) were classified as significantly differential metabolites.

### 2.5. cDNA Library Preparation and Transcriptome Sequencing

The aboveground parts of oat seedlings treated with CK, T0.02, and T0.1 were collected, with three biological replicates for each treatment concentration. Sample extraction and transcriptome sequencing were conducted by Shanghai Bioprofile Biotechnology Co., Ltd. Prior to sequencing, RNA purity (OD260/280, OD260/230) was assessed using a Nanodrop spectrophotometer (Thermo Fisher Scientific Inc., Waltham, MA, USA), and RNA integrity was evaluated by analyzing the RNA fragment length with an Agilent 2100 Bioanalyzer (Agilent Technologies, Santa Clara, CA, USA), ensuring that the high-quality library standards were met. The qualified RNA was enriched into mRNA using Oligo (dT) magnetic beads (New England Biolabs, Ipswich, MA, USA), followed by fragmentation into segments of approximately 300 bp via ion disruption. These fragments served as templates for cDNA synthesis, which was subsequently amplified and enriched through PCR to obtain a cDNA library. The library underwent quality checks using the Agilent 2100 Bioanalyzer. After RNA extraction, purification, and library construction, the libraries were subjected to paired-end (PE) sequencing utilizing second-generation sequencing technology based on the Illumina sequencing platform. To generate high-quality analyses, it is essential to filter raw sequencing data to produce clean datasets. The upgraded version of TopHat2, specifically the HISAT2 (http://ccb.jhu.edu/software/hisat2/index.shtml, accessed on 1 August 2024) software, was used to align the filtered reads to the reference genome (https://graingenes.org/GG3/graingenes-downloads/pepsico-oat-ot3098-v2-files-2021, accessed on 1 August 2024), resulting in mapped reads. Throughout this process, it is crucial to evaluate sequencing saturation, gene coverage, and the quality of reads across various regions of the reference genome. Differential expression analysis was conducted between treatment groups using DESeq (v1.39.0), applying the established criteria for screening DEGs: |log2FoldChange| > 1 and a significance *p*-value < 0.05. Finally, an enrichment analysis of KEGG pathways for the DEGs was performed.

### 2.6. qRT-PCR Analysis

Gene-specific primers were designed using Primer Premier 5 software and synthesized by BGI. The details of the primers utilized are provided in Supplementary Table S6. The qRT-PCR analysis was conducted on the Bio-Rad iQ5 real-time fluorescence quantitative PCR platform. The qRT-PCR reaction commenced with denaturation at 95 °C for 30 s, followed by 40 cycles of amplification at 95 °C for 5 s and 60 °C for 30 s. Subsequently, a melting curve step was performed at 95 °C for 15 s, 60 °C for 50 s, and 95 °C for 15 s. The melting curve analysis was employed to confirm the specificity of the primers. Actin was utilized as the reference gene for the expression of the AQP gene family in different oat tissues in oat [50], and used for the dynamic gene expression patterns of pollen abortion in a male sterile line of *Avena sativa* at different developmental stages [51]. qRT-PCR data were normalized with respect to the control treatment (CK). Each gene was subjected to three technical replicates and three biological replicates. The 2^−△△Ct^ [52] method was employed for data analysis.

### 2.7. Statistical Analysis

All data analyzed in this study are derived from three independent biological replicates, encompassing RNA sequencing and RT-qPCR analyses. Statistical analyses and graphical representations were conducted using Microsoft Excel 2021 and IBM SPSS Statistics 27, with results expressed as means ± SEM. Bars labeled with different lowercase letters indicate significant differences determined by one-way ANOVA followed by Duncan’s multiple range test (*p* < 0.05). Correlation analyses were performed on metabolites and genes associated with the phenylpropanoid and flavonoid biosynthetic pathways. Furthermore, the correlations between physiological and biochemical indices and metabolites were examined, as well as the relationships between metabolites within these pathways and selenium content in leaves. All correlation heatmaps were generated using the OmicShare tool (https://www.omicshare.cn, accessed on 1 April 2025). Three asterisks (***) denote a significance level of *p* < 0.001, two asterisks (**) denote a significance level of *p* < 0.01, and one asterisk (*) denotes a significance level of *p* < 0.05.

## 3. Results

### 3.1. Effect of Oat Seedlings on Morphology and Physiological Indexes Under Na_2_SeO_3_ Treatment

Selenium significantly influences the growth of oat seedlings. A series of treatments with varying concentrations of Na_2_SeO_3_ (CK, 0.01, 0.02, 0.05, 0.075, and 0.1 g/kg) were administered to the oat seedlings. The results from phenotypic and physiological assessments indicated that a Na_2_SeO_3_ concentration of 0.02 g/kg enhanced seedling growth, while a concentration of 0.1 g/kg led to yellowing of the seedling leaves and inhibited growth. Notable differences in physiological traits were observed among the CK, T0.02, and T0.1 treatments. In all subsequent experiments, samples treated with these three concentrations were selected for further analysis and comparison of metabolomic and transcriptomic results (Figure S1).

**Figure 1:**
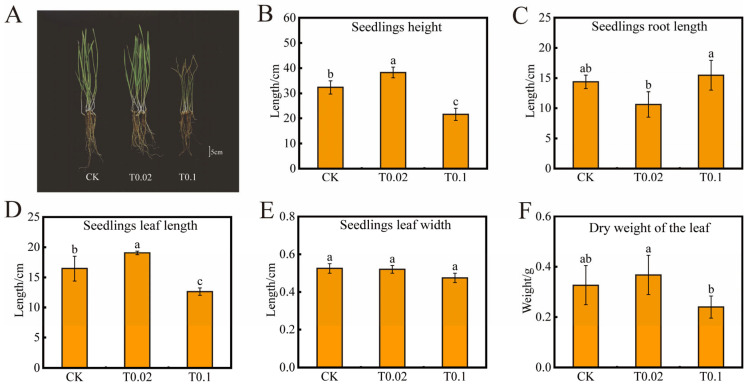
Effects of oat seedlings with different selenium treatments. (**A**) Phenotypes. (**B**) Seedlings height. (**C**) Seedlings’ root length. (**D**) Seedlings’ leaf length. (**E**) Seedlings’ leaf width. (**F**) Dry weight of the leaf. The bars with different lowercase letters represent significant differences based on one-way ANOVA with Duncan’s multiple range test (*p* < 0.05).

The T0.02 selenium treatment promoted the growth of oat seedlings, while the T0.1 selenium treatment exhibited an inhibitory effect, leading to wilting, senescence, and yellowing of the leaves (Figure 1A). The growth parameters of oat seedlings include plant height (Figure 1B), root length (Figure 1C), leaf length (Figure 1D), leaf width (Figure 1E), and leaf dry weight (Figure 1F). Compared to the control (CK) treatment, both plant height and leaf length initially increased before decreasing. The T0.02 treatment resulted in significant increases of 18.36% and 15.81% in plant height and leaf length, respectively (*p* < 0.05). Conversely, the T0.1 treatment led to significant reductions of 33.24% and 23.25% in these parameters (*p* < 0.05). No significant changes were observed in root length, leaf width, and root weight.

**Figure 2:**
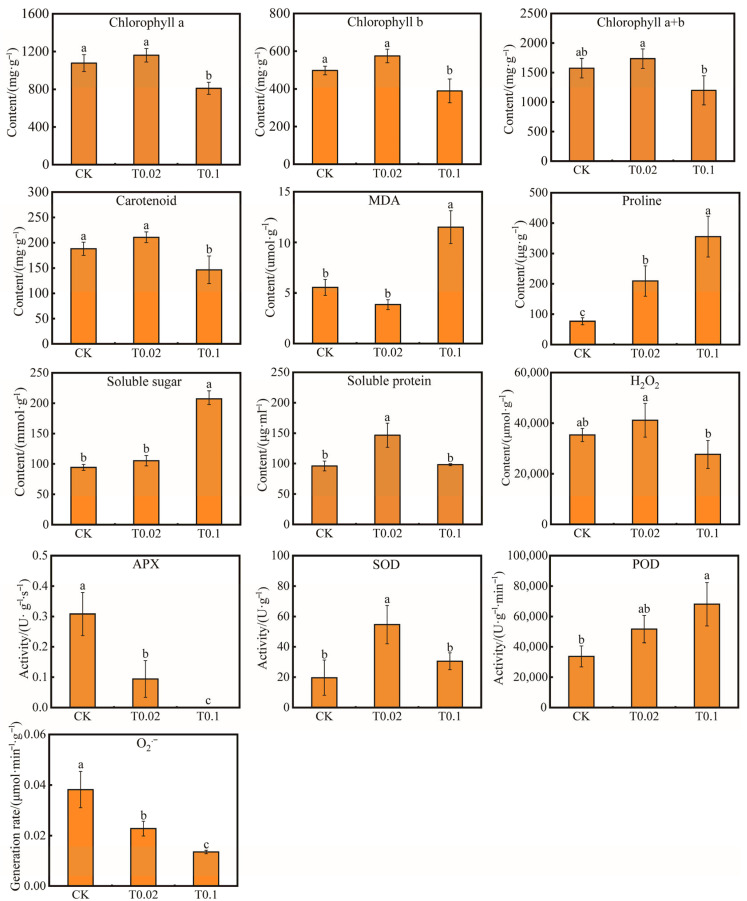
Effects of physiological indices of oat seedlings with different selenium treatments. The bars with different lowercase letters represent significant differences based on one-way ANOVA with Duncan’s multiple range test (*p* < 0.05).

Compared to the CK treatment, the soluble protein content and SOD activity significantly increased by 52.48% and 178.01% under T0.02 selenium treatment, respectively (*p* < 0.05, Figure 2). In contrast, the contents of chlorophyll a, chlorophyll b, and carotenoids decreased by 24.82%, 21.78%, and 22.12% under T0.1 selenium treatment (*p* < 0.05, Figure 2), respectively. Under T0.1 treatment, the levels of MDA, proline, soluble sugar, and POD activity also significantly increased (Figure 2). Specifically, under T0.1 selenium treatment, the contents of MDA, proline, soluble sugar, and the activity of POD increased by 107.11%, 362.81%, 121.72%, and 102.13% (*p* < 0.05, Figure 2), respectively. Conversely, the O_2_^−^ and APX activity decreased with increasing selenium concentration, showing reductions of 64.40% and 55.29% under T0.1 selenium treatment compared to CK treatment (*p* < 0.05, Figure 2), respectively. The contents of chlorophyll a + b and the hydrogen peroxide (H_2_O_2_) exhibited no significant changes.

### 3.2. Selenium Content and Mobility Coefficient in Oat Seedlings Under Na_2_SeO_3_ Treatment

**Figure 3:**
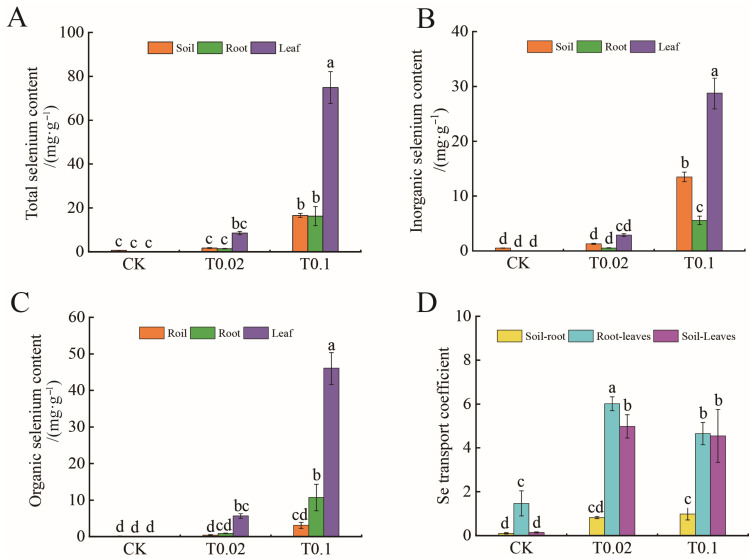
Selenium content and translocation factor in soil and oat seedlings with different selenium treatments. (**A**) Total selenium content. (**B**) Inorganic selenium content. (**C**) Organic selenium content. (**D**) Selenium transfer coefficient. The bars with different lowercase letters represent significant differences based on one-way ANOVA with Duncan’s multiple range test (*p* < 0.05).

This study examined the concentrations of total selenium, inorganic selenium, and organic selenium in soil and oat seedlings (Figure 3). Under the T0.02 treatment, the concentrations of total selenium and inorganic selenium in both the soil and oat seedlings increased compared to the control, although these differences did not reach statistical significance (Figure 3). In contrast, under the T0.1 treatment, the total selenium content in the soil, oat seedling roots, and leaves increased by 24.23, 231.59, and 770.97 times, respectively (*p* < 0.05, Figure 3A). Furthermore, the inorganic selenium content in the soil, oat seedling roots, and leaves increased by 24.82, 213.65, and 879.09 times, respectively (*p* < 0.05, Figure 3B). Additionally, the organic selenium content in the roots and leaves of oat seedlings increased by 242.18 and 719.34 times, respectively (*p* < 0.05, Figure 3C).

The mobility coefficient of selenium indicates its ability to migrate between soil and plant. Significant differences in the mobility coefficients of selenium in different parts of the oat seedlings were observed (Figure 3D). Under T0.02 treatment, the root-to-leaf mobility coefficient (6.01) was significantly higher than that of soil-to-root (0.83) and soil-to-leaf (4.98) (Figure 3D). Under treatment T0.1, the migration coefficient from root to leaf (4.65) or from soil to leaf (4.55) was significantly higher than the migration coefficient from soil to root (0.99) (Figure 3D). Therefore, selenium treatment significantly improved the root-to-leaf mobility coefficient.

### 3.3. Metabolomic Analysis of Oat Seedlings Under Na_2_SeO_3_ Treatment

**Figure 4:**
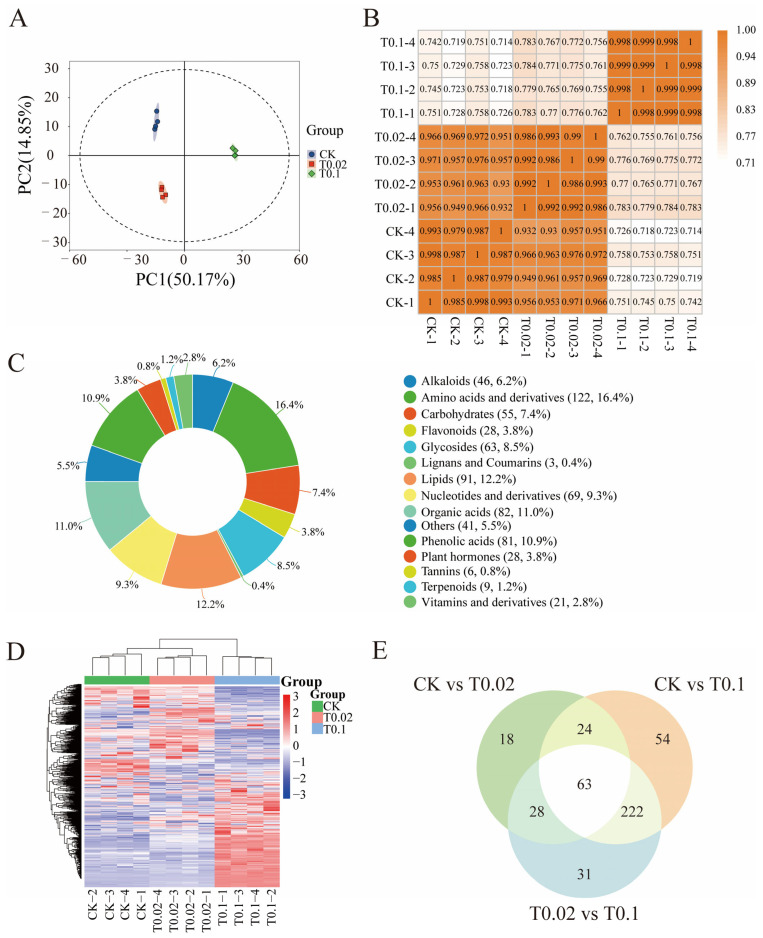
Quality control of metabolomic data and changes in differentially accumulated metabolites (DAMs) levels in oat seedlings with different selenium treatments. (**A**) Principal component analysis of DAMs. (**B**) Correlation analysis of DAMs. (**C**) Types and proportions of annotated metabolites. (**D**) Heatmap of all the identified metabolites, ranging from low (blue) to high (red). (**E**) Venn diagram of differential metabolites. The overlap represents the proportion of metabolites shared by groups, while the non-overlap represents the proportion of metabolites specific to the group.

In this study, non-targeted metabolomics (LC-MS) was employed to analyze the aboveground parts of oat seedlings, revealing a variety of metabolites associated with selenium stress. This analysis elucidates the response mechanisms of the aerial parts under selenium stress. Based on the metabolomics data from oat seedlings subjected to different treatments, the PCA results indicated that the explained variances for PC1 and PC2 were 50.17% and 14.85%, respectively (Figure 4A). These results demonstrated significant differences among samples from various treatments along both PC1 and PC2 (Figure 4A). Furthermore, correlation analysis (R > 0.70) indicated a higher correlation within samples than between different samples, thereby confirming the stability and reliability of the metabolomic data obtained (Figure 4B).

A total of 745 metabolites were detected, including 122 amino acids and derivatives, accounting for 16.4% of metabolites, 91 lipids for 12.2%, 82 organic acids for 11%, 81 phenolic acids for 10.9%, 69 nucleotides and derivatives for 9.3%, 63 glycosides for 8.5%, 55 carbohydrates for 7.4%, 46 alkaloids for 6.2%, and 28 flavonoids for 3.8% (Figure 4C, Table S1). The cluster heat map indicated significant differences in the abundance of the 745 metabolites under various selenium treatments (Figure 4D). Approximately half of the metabolites under the T0.1 treatment exhibited an increase in enrichment abundance, while the other half showed a decrease (Figure 4D). To further evaluate the changes in metabolites in oat seedlings under selenium treatment, metabolites were selected based on multivariate statistical analysis with a VIP > 1 and univariate statistical analysis with a *p* value < 0.05, identifying them as significantly different (Figure 4E). Venn diagram analysis revealed that 63 differentially accumulated metabolites (DAMs) were shared among the CK, T0.02, and T0.1 treatments. The specific metabolites identified in the comparisons of CK vs. T0.02, CK vs. T0.1, and T0.02 vs. T0.1 were 18, 54, and 31, respectively (Figure 4E, Tables S2 and S3). A total of 24 metabolites might represent general metabolites in oat response to T0.02 and T0.1 treatments, and are not involved in the phenylpropanoid and flavonoid biosynthesis pathways.

### 3.4. KEGG Pathway Analysis of DAMs in Oat Seedlings Under Na_2_SeO_3_ Treatment

**Figure 5:**
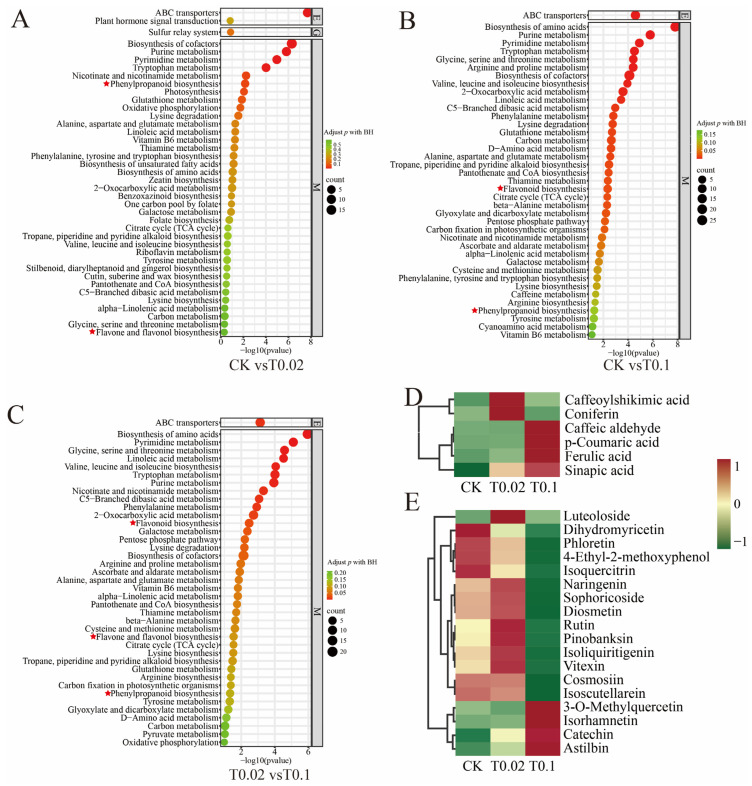
KEGG pathway analysis of the top 40 DAMs in oat seedlings with different selenium treatments. (**A**) KEGG analysis of differential metabolites in CK vs. T0.02. (**B**) KEGG analysis of differential metabolites in CK vs. T0.1. (**C**) KEGG analysis of differential metabolites in T0.02 vs. T0.1. (**D**) Heat map of 6 DAMs associated with the Phenylpropanoid biosynthesis pathway. (**E**) Heat map of 18 DAMs associated with flavonoid biosynthesis pathways. The color indicates the level of accumulation of each metabolite, ranging from low (green) to high (red).

Based on the KEGG database, we conducted a functional annotation of DAMs to identify key metabolic pathways associated with selenium treatment in the aboveground parts of oat seedlings (Figure 5). We selected the top 40 metabolic pathways with the lowest *p*-values in oat seedlings. The results indicated that in the CK vs. T0.02 comparison group, phenylpropanoid biosynthesis, as well as flavone and flavonol biosynthesis, were significantly enriched (Figure 5A, Table S4). In the CK vs. T0.1 group, both flavonoid and phenylpropanoid biosynthesis were significantly enriched (Figure 5B, Table S4). In the T0.02 vs. T0.1 group, flavonoid, flavone, and flavonol, and phenylpropanoid biosynthesis showed significant enrichment (Figure 5C, Table S4).

We applied the criteria of fold change (FC) > 1, *p* value < 0.05, and VIP > 1 to identify DAMs. A total of six DAMs associated with phenylpropanoid biosynthesis and 18 DAMs linked to flavonoid biosynthesis were identified (Figure 5D,E, Table S5). Compared with the CK, in the phenylpropanoid biosynthesis pathway (Figure 5D), three compounds (Caffeoylshikimic acid, Coniferin, and Sinapic acid) exhibited elevated levels under T0.02 treatment, and four compounds (Caffeyl-aldehyde, p-Coumaric acid, Ferulic acid, and Sinapic acid) demonstrated increased levels under T0.1 treatment. In the flavonoid biosynthesis pathway (Figure 5E), eight compounds (Luteoloside, Naringenin, Sophoricoside, Diosmetin, Rutin, Pinobanksin, Isoliquiritigenin, and Vitexin) showed higher concentrations under T0.02 treatment, while four compounds (3-O-Methylquercetin, Isorhamnetin, Catechin, and Astilbin) exhibited increased levels under T0.1 treatment. Overall, under T0.02 treatment, 33.33% of phenolic acid metabolites and 44.45% of flavonoid metabolites were increased. Under T0.1 treatment, 66.67% of phenolic acid metabolites and 22.22% of flavonoid metabolites were increased.

### 3.5. Transcriptomic Analysis of Oat Seedlings Under Na_2_SeO_3_ Treatment

**Figure 6:**
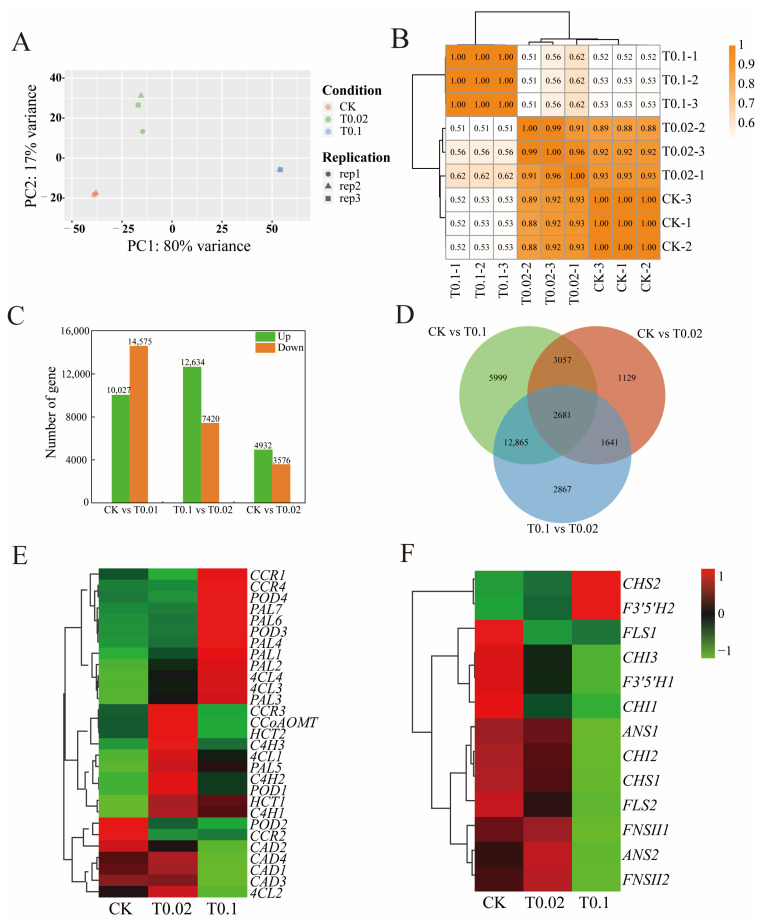
Transcriptome data of oat seedlings with different selenium treatments. (**A**) Principal component analysis of DEGs. (**B**) DEGs correlation heat map. (**C**) The numbers of upward and downward DEGs. (**D**) Venn diagram of DEGs. The overlap represents the proportion of genes shared with groups, while the non-overlap represents the proportion of genes that are unique to the group. (**E**) Heat map of DEGs associated with phenylpropanoid biosynthesis pathway. (**F**) Heat map of DEGs associated with flavonoid biosynthesis pathways. The color indicates the level of accumulation of each transcript, ranging from low (green) to high (red).

Transcriptome analysis was conducted to assess gene expression changes in oat seedlings subjected to CK, T0.02, and T0.1 treatments. After filtering out low-quality reads, a total of 48,946,142 to 82,466,668 clean reads were obtained. The Q30 percentage ranged from 96.22% to 97.26%, indicating that the transcriptome sequencing data are of high quality (Table S6). PCA analysis revealed significant differences in gene expression across varying selenium concentrations (Figure 6A), while the correlation heat map indicated a correlation coefficient between 0.88 and 1.00 for all three treatment groups (Figure 6B). Furthermore, a difference was defined as |log2FoldChange| > 1, with a significance *p*-value < 0.05 used as the screening criterion for differential genes among the three treatment groups. A total of 24,602 differentially expressed genes (DEGs) were identified in CK vs. T0.02 (10,027 upregulated and 14,575 downregulated), 20,054 DEGs (12,634 upregulated and 7420 downregulated) in CK vs. T0.1, and 8508 DEGs (4932 upregulated and 3576 downregulated) in T0.1 vs. T0.02 (Figure 6C). A Venn diagram was constructed to analyze the DEGs across different treatments, revealing 5999 unique DEGs in CK vs. T0.1, 1129 in CK vs. T0.02, and 2867 in T0.1 vs. T0.02, with 2681 DEGs shared among CK, T0.1, and T0.02 (Figure 6D).

### 3.6. DEGs in the Phenylpropanoid and Flavonoid Biosynthesis in Oat Seedlings

Cluster analysis of DEGs involved in the phenylpropanoid biosynthesis pathway under varying selenium treatments revealed significant variations in gene expression with increasing selenium concentration (Figure 6E). In this study, we identified 29 differential genes associated with the phenylpropanoid biosynthesis pathway, including *PAL* (7), *C4H* (3), *4CL* (4), *HCT* (2), *CCR* (4), *CAD* (4), *POD* (4), and *CCoAOMT* (1) *(*Figure 6E, Table S7). Notably, compared to the CK treatment, the genes *CCR3*, *CCoAOMT*, *HCT2*, *C4H3*, *4CL1*, *PAL5*, *C4H2*, *POD1*, *HCT1*, *C4H1*, and *4CL2* exhibited high expression levels under the T0.02 treatment (Figure 6E). Conversely, genes *CCR1*, *CCR4*, *POD4*, *PAL7*, *PAL6*, *POD3*, *PAL4*, *PAL1*, *PAL2*, *4CL4*, *4CL3*, and *PAL3* showed high expression levels under the T0.1 treatment, while *CAD2*, *CAD4*, *CAD1*, *CAD3*, and *4CL2* were expressed at lower levels (Figure 6E).

Thirteen DEGs associated with the flavonoid biosynthesis pathway were identified, including *CHS* (2), *CHI* (3), *F3′5′H* (2), *ANS* (2), *FNSII* (2), and *FLS* (2) (Figure 6F, Table S7). The results indicated that, compared to the CK treatment, under the T0.02 treatment, *ANS2* and *FNSII2* were highly expressed, whereas *FLS1* was expressed at low levels (Figure 6F). Under the T0.1 treatment, *CHS2* and *F3′5′H2* exhibited high expression levels, whereas *CHI3*, *F3′5′H1*, *CHI1*, *ANS1*, *CHI2*, *CHS1*, *FLS2*, *FNSII1*, *FNSII2*, and *ANS2* were expressed at lower levels (Figure 6F).

### 3.7. Verification of DEGs in Phenylpropanoid and Flavonoid Metabolism in Oat Seedlings by RT-qPCR

**Figure 7:**
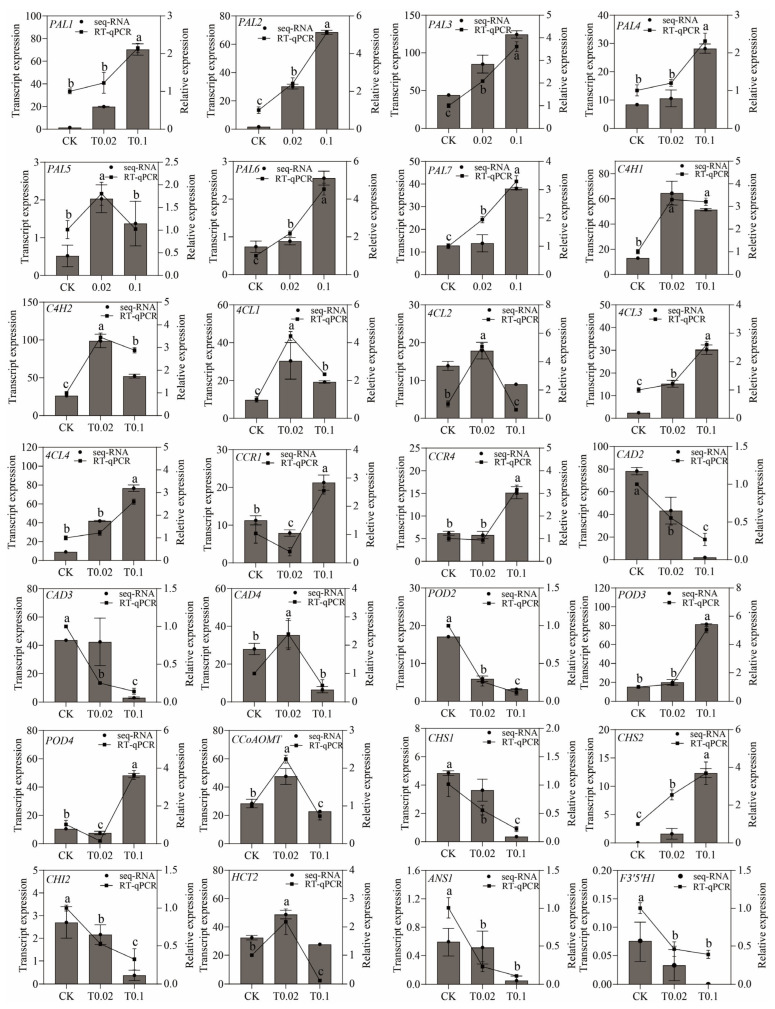
The expression of genes in the phenylpropanoid and flavonoid biosynthesis pathway was analyzed by seq-RNA and RT-qPCR. The column chart reflects the FPKM value of the transcript expression, while the line chart reflects the relative gene expression by RT-qPCR method. The relative gene expression was calculated using the 2^−ΔΔCt^ method. The vertical bars in the line chart indicate the mean ± standard deviation, with *n* = 3. Line plots marked with different lowercase letters signify significant differences using one-way analysis of variance (ANOVA) followed by Duncan’s multiple range test (*p* < 0.05).

A total of 28 genes were selected from the 42 DEGs within the phenylpropanoid and flavonoid biosynthetic pathway for validation via RT-qPCR, with the objective of confirming the reliability and accuracy of the RNA-seq data (Table S8). The *Actin* gene was utilized as an endogenous reference to normalize the expression levels of the target genes. The results indicated that the relative expression of the genes assessed by RT-qPCR was consistent with the transcriptional expression obtained from RNA-seq data, thereby enhancing the authenticity and reliability of the transcriptomic data (Figure 7).

The relative expression levels of the *PAL1*, *PAL2*, *PAL3*, *PAL4*, *PAL6*, *PAL7*, *4CL3*, *4CL4*, *POD3*, and *CHS2* genes exhibited an upward trend, while the relative expression levels of *CAD2*, *CAD3*, *POD2*, *CHS1*, *CHI2*, *ANS1*, and *F3′5′H* genes showed a downward trend. The expression levels of *PAL5*, *C4H1*, *C4H2*, *4CL1*, *4CL2*, *CAD4*, *CCoAOMT*, and *HCT2* genes showed high relative expression under T0.02 treatment (Figure 7).

### 3.8. Correlation Analysis of Physiological and Biochemical Indicators, Gene Expression, Metabolites

**Figure 8:**
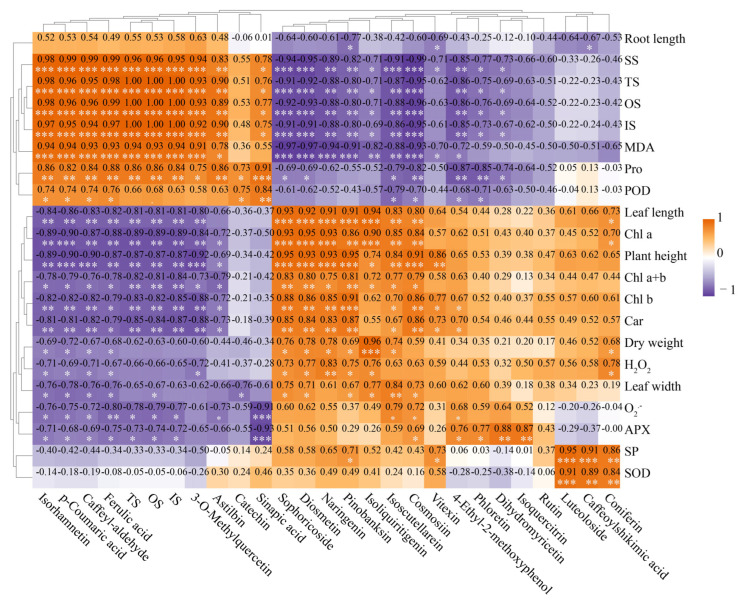
Physiological and biochemical indices in the biosynthesis pathways of phenylpropanoid and flavonoids, and the correlation between selenium content and metabolite content. Orange and purple indicate positive and negative correlations, respectively. The significance level is expressed as follows: *: *p* < 0.05; **: *p* < 0.01, ***: *p*< 0.001.

A comprehensive analysis of the physiological and biochemical indicators, along with the metabolites involved in the phenylpropanoid and flavonoid biosynthesis pathway and the selenium content in oat leaves, revealed a total of 148 positive correlations and 149 negative correlations (*p* < 0.05, *p* < 0.01, *p* < 0.001, Figure 8). The levels of soluble sugars (SS) and MDA exhibited significant positive correlations with nine compounds, while plant height, leaf length, and pigment content of the seedlings showed significant negative correlations with them (*p* < 0.05, *p* < 0.01, *p* < 0.001, Figure 8). The levels of SS and MDA indicated significant negative correlations with nine compounds, while plant height, leaf length, and pigment content of the seedlings showed significant positive correlations with seven of them (*p* < 0.05, *p* < 0.01, *p* < 0.001, Figure 8). Additionally, the soluble protein (SP) content and SOD activity demonstrated significant positive correlations with Luteoloside, Caffeoylshikimic acid, and Coniferin (*p* < 0.01, *p* < 0.001, Figure 8).

Notably, the total selenium, organic selenium, and inorganic selenium contents in leaves exhibited significant positive correlations with seven metabolites (*p* < 0.05, *p* < 0.01, *p* < 0.001, Figure 8), while exhibited significant negative correlations with 10 metabolites (Cosmosiin, Sophoricoside, Diosmetin, et al.) (*p* < 0.05, *p* < 0.01, *p* < 0.001, Figure 8). These findings indicate that the selenium content in seedling leaves may directly or indirectly influence the accumulation of 17 metabolites.

H_2_O_2_ content was positively correlated with the contents of five flavonoids and one phenylpropanoid compound (Coniferin). O_2_^−^ content was positively correlated with those of three flavonoids (*p* < 0.05, *p* < 0.01, Figure 8). H_2_O_2_ content was negatively correlated with that of two flavonoids and three phenylpropanoid compounds, and O_2_^−^ content was negatively correlated with that of two flavonoid compounds and four phenylpropanoid compounds. Above all, the contents of ROS were mainly positively correlated with those of flavonoid compounds, and negatively correlated with those of phenylpropanoid compounds.

**Figure 9:**
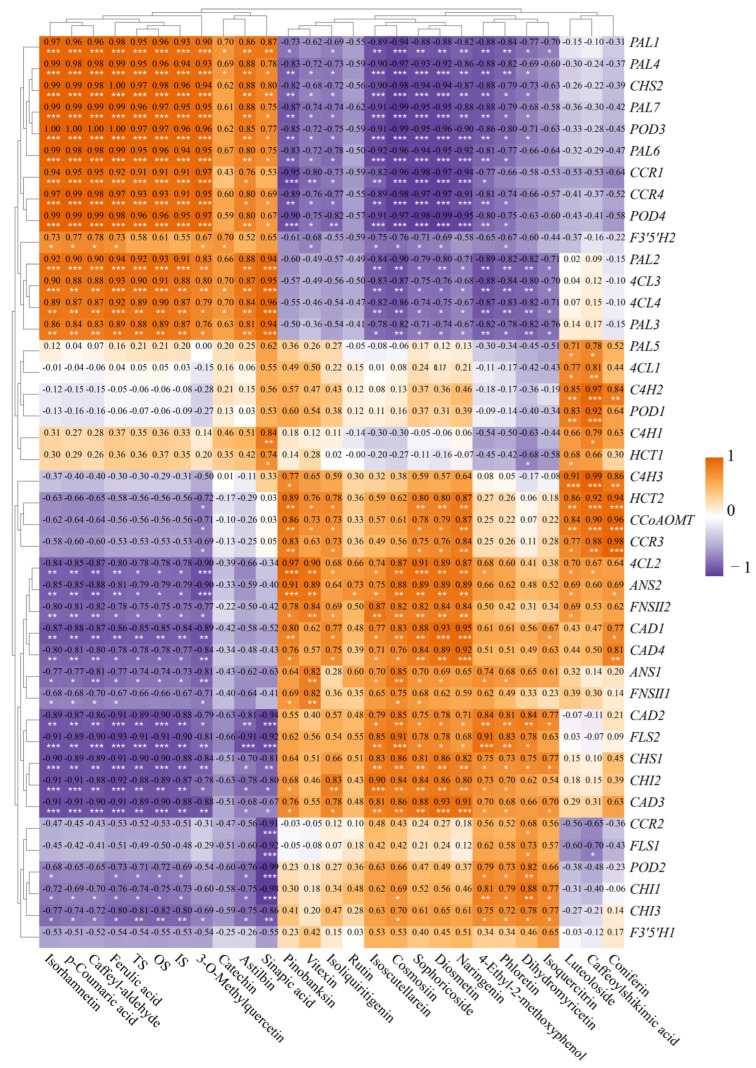
Correlation between gene expression and metabolites in phenylpropanoids and flavonoid biosynthesis pathways. Orange and purple indicate positive and negative correlations, respectively. The significance level is expressed as follows: *: *p* < 0.05; **: *p* < 0.01, ***: *p* < 0.001.

In the phenylpropanoid and flavonoid biosynthesis pathway, a correlation analysis involving 42 genes and 27 compounds (including selenium content) in leaves revealed 300 positive and 271 negative correlations (*p* < 0.05, *p* < 0.01, *p* < 0.001, Figure 9). Among these, the expressions of 13 genes (*PAL1* to *PAL3* from top to bottom in Figure 9, except F3′5′H2) exhibited significant positive correlations with the contents of nine compounds (*p* < 0.05, *p* < 0.01, *p* < 0.001). Expressions of 12 genes (*4CL2* to *CAD3* from top to bottom in Figure 9, except FNSII1) demonstrated significant positive correlations with contents of 7–10 metabolites (*p* < 0.05, *p* < 0.01, *p* < 0.001), alongside significant negative correlations with those of 8–10 compounds in leaves (*p* < 0.05, *p* < 0.01, *p* < 0.001, Figure 9). In summary, over 60% of the genes (25/42) in the phenylpropanoid and flavonoid biosynthesis pathway were associated with the accumulation of about 74% (20/27) compounds in oat leaves. There were 13 major genes (including *PAL1*, *PAL4*, *CHS2*, *PAL7*, *POD3*, *PAL6*, *CCR1*, *CCR4*, *POD4*, *PAL2*, *4CL3*, *4CL4*, and *PAL3*) likely modulating the metabolism of phenylpropanoid and flavonoid biosynthesis pathway.

## 4. Discussion

Research indicates that selenium has a dual effect on the growth and development of plants: low concentrations promote development, while high concentrations inhibit it [53,54]. At a lower concentration (100 mg/kg), sodium selenite positively affects alfalfa by enhancing the activity of antioxidant enzymes [55]. However, excessive selenium reduces the content of photosynthetic pigments and chlorophyll fluorescence parameters in cucumber leaves [56]. Elevated selenium levels in plants disrupt metabolic processes, leading to phytotoxicity, which manifests as visual symptoms, such as leaf chlorosis and necrosis [57]. In this study, selenium exhibited similar effects on oats. Under low selenium treatment (T0.02), oat seedlings displayed vigorous growth. However, under high selenium treatment (T0.1), the plant height, leaf length, dry weight, and pigment content of oat seedlings significantly decreased. These changes resulted in developmental retardation, leaf chlorosis, and growth inhibition in oat seedlings, indicating that high selenium treatment damaged the chloroplast structure, affected the synthesis of photosynthetic pigments, and ultimately impaired the overall growth of oat seedlings.

MDA, soluble sugars, and soluble proteins are critical indicators for evaluating plant physiological metabolism and stress adaptation capabilities. Foliar application of selenium significantly increased the levels of soluble sugars and proteins in *Ginkgo biloba* leaves [14]. Under high selenium stress conditions, MDA levels in soybean roots and leaves were elevated [58]. In this study, soluble protein levels significantly increased under low selenium treatment, whereas both soluble sugar and MDA content significantly increased under high selenium treatment. This suggests that plants synthesize more metabolic enzymes and defense proteins under low selenium conditions, thereby enhancing their metabolic and adaptive capabilities. Conversely, under high selenium conditions, the extent of lipid peroxidation in cell membranes intensifies, leading to increased osmotic substance content and structural damage to cells [59]. Proline serves as an essential osmotic regulatory substance. Djanaguiraman et al. [60] demonstrated that selenium can induce proline accumulation in soybeans, thereby enhancing their stress resistance. In this study, the T0.1 treatment resulted in a greater accumulation of proline compared to the T0.02 treatment, indicating that plants regulate cellular osmotic pressure through proline accumulation to better adapt to selenium stress in high-selenium environments.

Previous studies have demonstrated that the activities of antioxidant enzymes in quinoa plants, including SOD, POD, and APX, significantly decrease at low concentrations of Na_2_SeO_3_·5H_2_O (2.5 and 5 mg/L) [61]. In corn (*Zea mays* L.), the activities of SOD, POD, and APX also decrease under high selenium treatment [62]. In this study, under low selenium treatment, SOD activity significantly increased, indicating its primary role; whereas under high selenium treatment, POD activity significantly increased, suggesting that oat seedlings may rely more on POD or non-enzymatic antioxidants (such as ascorbic acid and glutathione) to cope with oxidative stress, which may explain the lack of significant changes in SOD activity. The enhancement of POD’s antioxidant capacity under high selenium treatment may lead to a reduction in the generation rate of superoxide anions and APX activity, resulting in the efficient allocation of experimental antioxidant resources. Additionally, the H_2_O_2_ content did not exhibit significant changes, possibly due to the plant maintaining a dynamic balance through complex physiological and biochemical regulatory mechanisms. Conversely, previous studies have indicated that lignin and flavonoids may play a role in scavenging reactive ROS in the root tissues of Sophora alopecuroides, thereby mitigating damage caused by salt stress [63]. This study emphasizes that non-enzymatic antioxidants, such as flavonoids and phenylpropanoids, are effective in scavenging ROS, thereby alleviating the burden on antioxidant enzymes.

The application of selenate in cabbage (*Brassica oleracea var. capitata* L.) and sodium selenite in *Broussonetia Papyrifera* has been shown to significantly enhance the total selenium content [15,64]. Furthermore, exogenous selenium application in tea plants markedly increased both the total selenium and organic selenium content in tea leaves, while also promoting the accumulation of various secondary metabolites, thereby enhancing the nutritional value of tea [65]. The selenium content in buckwheat grains exhibited a positive correlation with the applied selenium concentration, increasing with higher selenium application rates [66]. In this study, high selenium treatment significantly elevated the total selenium, inorganic selenium, and organic selenium content in both roots and leaves. Additionally, the selenium content in leaves facilitated the accumulation of metabolites such as isorhamnetin, ferulic acid, and 3-O-methylquercetin. Notably, selenium treatment also significantly increased the translocation coefficient from roots to leaves.

Nano-selenium treatment significantly enhances the levels of phenylalanine, naringenin, and pinoresinol in peaches, thereby activating phenylpropanoid metabolism [67]. Additionally, this treatment notably increases the content of various substances, including phenylalanine and coniferaldehyde, in *Perilla frutescens* (L.), thereby improving their biochemical disorders and overall quality [68]. Furthermore, nano-selenium promotes the biosynthesis of flavonoids such as apigenin and rutin in celery [69]. In contrast, treatments using sodium selenate and selenium nanoparticles in tomatoes result in a reduction of β-carotene content, an increase in naringenin and chlorogenic acid levels, and a decrease in coumaric acid levels [70]. In this study, with an increase in selenium concentration, the contents of six metabolites (p-Coumaric acid, Ferulic acid, Sinapic acid, Isorhamnetin, Catechin, and Astilbin, Figure 5D,E) also increased. Notably, soluble sugar, MDA, and proline content exhibited significant positive correlations with Isorhamnetin, Caffeyl-aldehyde, Ferulic acid, 3-O-Methylquercetin, and Astilbin (Figure 8). We performed correlation analyses for all physiological indicators and all differential metabolites of phenylpropane and flavonoids, showing that hydrogen peroxide was positively correlated with six metabolites and negatively correlated with five metabolites (Sophoricoside, Diosmetin, Naringenin, Pinobanksin, Isoliquiritigenin, Isoscutellarein), which were significantly increased under T0.1 treatment (Figure 5D,E). These metabolites can directly scavenge ROS, resulting in a reduced rate of superoxide anion production. As previous studies have shown, benzothiazole (BTH) can delay senescence and maintain the quality of *Rosa roxburghii* fruit by modulating ROS metabolism and the phenylpropanoid pathway under low-temperature conditions [71]. Melatonin (MT) enhances the antioxidant capacity by activating both the ROS metabolism and phenylpropanoid pathway, thus maintaining the quality of fresh-cut *Gastrodia elata* [72].

**Figure 10:**
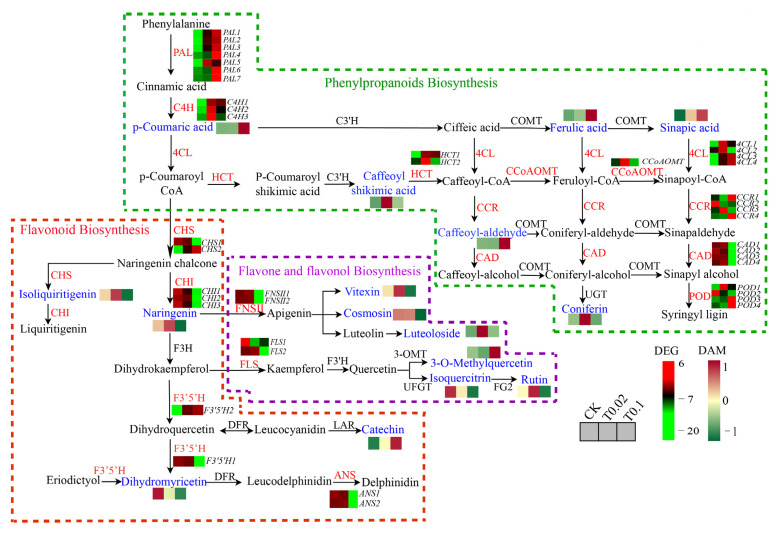
Integrated analysis of DEGs and DAMs in the phenylpropanoid biosynthesis pathway, including phenylpropane biosynthesis pathway, flavonoid biosynthesis pathway, flavonoid and flavonol biosynthesis pathway, in oat seedlings under selenium treatment. The blue font represents the differential metabolites, and the red font represents the differential genes. Rectangles indicate differential genes or differential metabolites, and the color of the rectangles indicates the regulation of genes or metabolites under selenium treatment. The accumulation level of DEG and DAM from low (green) to high (red). The blocks arranged from left to right correspond to the CK, T0.02, and T0.1 groups. The heatmap shows the normalized FPKM values, normalized by Z-score.

The cultivation of peanut (*Arachis hypogaea* L.) seedlings with sodium selenite significantly upregulated the expression of genes associated with the phenylpropanoid biosynthesis pathway, including *PAL*, *C4H*, and *CHS*, thereby alleviating selenite stress [73]. Selenium regulation notably induced the expression of *PAL*, *F3H*, *DFR*, *CHS*, and *C4H*-related genes in waxy maize (*Zea mays* L.) [74]. The application of nano-selenium in peppers exposed to cadmium significantly enhanced the accumulation of lignin-related genes (such as *PAL*, *CAD*, *4CL*) and metabolites (including sinapyl alcohol, phenylalanine, and p-coumaryl alcohol), thus maintaining the integrity of root cell walls [75]. Furthermore, selenium enhances the expression of seven key structural genes involved in flavonoid biosynthesis in bread wheat (*Triticum aestivum* L.), specifically comprising two *TaF3Hs*, two *TaDFRs*, one *TaF3′5′H*, one *TaOMT*, and one *TaANR* [76]. Based on the conjoint analysis of differential metabolites and genes, the phenylpropanoid and flavonoid biosynthesis pathways in oat seedlings under selenium treatment were constructed (Figure 10). The expression levels of the *PAL1*, *PAL2*, *PAL3*, *PAL4*, *PAL6*, and *PAL7* genes were significantly positively correlated with the metabolites p-Coumaric acid, Ferulic acid, and Sinapic acid, indicating that these genes promote the accumulation of these metabolites (Figure 5D and Figure 10). The *4CL1*, *4CL2*, and *HCT2* genes also facilitated the accumulation of Caffeoylshikimic acid (Figure 5D and Figure 10). Furthermore, the *CCR1* and *CCR4* genes contributed to the accumulation of Caffeyl-aldehyde (Figure 5D and Figure 10). The *CHI2* gene promoted the accumulation of Isoliquiritigenin and Naringenin, while inhibiting their further accumulation. The *FNSII1* and *FNSII2* genes enhanced the accumulation of Vitexin, Cosmosiin, and Luteoloside (Figure 5E and Figure 10). Additionally, the *F3′5′H2* gene facilitated the accumulation of Catechin. The expression of 4 upstream genes, *PAL1*, *PAL4*, *4CL3*, and *4CL4*, exhibited a positive correlation with Catechin accumulation, with no negative regulatory genes identified, leading to a significant increase in their levels (Figure 5E and Figure 10). Moreover, the expressions of nine upstream genes *PAL1*, *PAL4*, *CHS2*, *PAL7*, *PAL6*, *PAL2*, *4CL3*, *4CL4*, and *PAL3* were positively correlated with 3-O-Methylquercetin, resulting in a substantial increase in its accumulation (Figure 5E and Figure 10).

The results indicate that selenium treatment significantly influences gene expression and metabolite accumulation in the phenylpropanoid and flavonoid biosynthesis pathways in oat seedlings. These findings offer valuable insights into the regulatory mechanisms that govern phenylpropanoids and flavonoids.

## 5. Conclusions

This study demonstrated a significant concentration-dependent effect of selenium on the growth of oat seedlings. The T0.02 treatment promoted the growth of oat seedlings. The pigment content was decreased significantly under T0.1 treatment, but the content of MDA, proline, and soluble sugar was increased significantly. Under the T0.02 treatment, the selenium transfer coefficient from root to leaf was the largest. Metabolomic analyses revealed six differential metabolites in the phenylpropanoid pathway, and 18 differential metabolites in the flavonoid pathway were screened. Nine key genes (*PAL1*, *PAL4*, *CHS2*, *PAL7*, *POD3*, *PAL6*, *CCR1*, *CCR4* and *POD4*) were found to regulate phenylpropane and flavonoid metabolism. This study provided scientific evidence for elucidating the molecular regulatory mechanisms of phenylpropanoid and flavonoid biosynthesis pathways in selenium-treated oat seedlings.

## Data Availability

The original contributions presented in this study are included in the article, supplementary material and https://dataview.ncbi.nlm.nih.gov/object/PRJNA1266609?reviewer=8fir4cnj5893k1sb1dof3iihc4, accessed on 1 May 2025. Further inquiries can be directed to the corresponding author.

## Supplementary Materials

The following supporting information can be downloaded at: https://www.mdpi.com/article/10.3390/biology14091131/s1, Figure S1: The phenotype of the seedlings under the treatments with different concentrations of Na_2_SeO_3_. Table S1: Annotated to all metabolites in oat seedlings under Na_2_SeO_3_ treatments. Table S2: Differential metabolites in oat seedlings under Na_2_SeO_3_ treatments between two groups. Table S3: 24 The DAMs shared between T0.02 and T0.1 treatments with respect to CK. Table S4: KEGG enrichment of metabolites under Na_2_SeO_3_ treatments. Table S5: 24 DAMs in the phenylpropane and flavonoid biosynthesis pathways in oat seedlings under Na_2_SeO_3_ treatments. Table S6: Statistical analysis of the transcript data in oat seedlings under Na_2_SeO_3_ treatments. Table S7: Potential candidate 42 DEGs involved in phenylpropane and flavonoid biosynthesis. Table S8: The information of primers used in the qRT-PCR experiment.

## Abbreviations

The following abbreviations are used in this manuscript:

TS	Total selenium content
OS	Organic selenium content
IS	Inorganic selenium content
MDA	Malondialdehyde
H_2_O_2_	Hydrogen peroxide
APX	Ascorbate peroxidase
SOD	Superoxide dismutase
O_2_^−^	Superoxide anion radical
*PAL*	Phenylalanine ammonia-lyase
*C4H*	Trans-cinnamate 4-monooxygenase
*4CL*	4-coumarate--CoA ligase
*CCR*	Cinnamoyl-CoA reductase
*CAD*	Cinnamyl alcohol dehydrogenase
*POD*	Peroxidase
*CCoAOMT*	Caffeoyl-CoA O-methyltransferase
*CHS*	Chalcone synthase
*CHI*	Chalcone isomerase
*ANS*	Anthocyanidin synthase
*F3′5′H*	Flavonoid 3′,5′-hydroxylase
*HCT*	Hydroxycinnamoyl transferase
*FLS*	Flavonol synthase
*FNS**II*	Flavone synthase II
*DFR*	Dihydroflavonol 4-reductase
*COMT*	Caffeic acid 3-O-methyltransferase
*UGT*	Uridine Diphosphate Glycosyltransferase
*F3′H*	Flavonoid 3′-monooxygenase
*F3H*	Naringenin 3-dioxygenase
*3-OMT*	Flavonol 3-O-methyltransferase
*UFGT*	Flavonol 3-O-glucosyltransferase
*FG2*	Flavonol-3-O-glucoside L-rhamnosyltransferase
*LAR*	Leucoanthocyanidin reductase
