# Supplementary material for: Molecular Regulation of Phenylpropanoid and Flavonoid Biosynthesis Pathways Based on Transcriptomic and Metabolomic Analyses in Oat Seedlings Under Sodium Selenite Treatment
Source: Biology (Basel). 2025 Aug 26;14(9):1131. doi: 10.3390/biology14091131 (PMC12467305; doi:10.3390/biology14091131)
Supplement: Supplementary file 1 [file biology-14-01131-s001.zip › Figure.pdf]

Figure S1 The phenotype of the seedlings under the treatments with different concentrations of Na_2SeO_3.

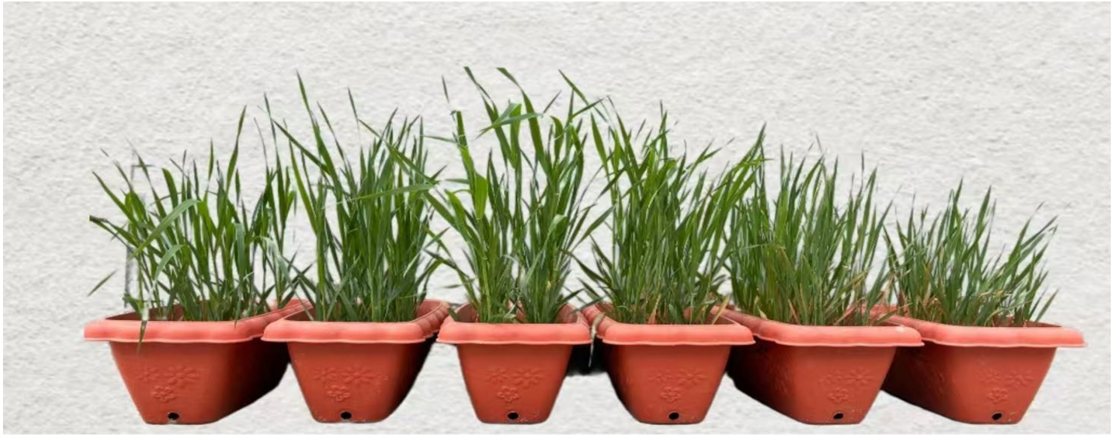

0 g·kg^{-1} 0.01 g·kg^{-1} 0.02 g·kg^{-1} 0.05 g·kg^{-1} 0.075 g·kg^{-1} 0.1 g·kg^{-1}
